# Painless thyroiditis following mRNA vaccination for COVID-19

**DOI:** 10.1007/s42000-021-00346-7

**Published:** 2022-01-15

**Authors:** Nobuhiko Nakaizumi, Shuji Fukata, Takashi Akamizu

**Affiliations:** Department of Internal Medicine, Kuma Hospital, Kuma Byoin, Kobe, Hyogo Japan

Recently, cases of subacute thyroiditis and Graves’ disease following coronavirus disease 2019 (COVID-19) vaccine immunization have been reported [1–4]. Herein, we present two cases of painless thyroiditis (PT) after the administration of the mRNA COVID-19 vaccine.

## Case 1

A 38-year-old woman presented with palpitations 17 days after the first dose of the COVID-19 Pfizer-BioNTech vaccine and was referred to the Department of Internal Medicine, Kuma Hospital, Kobe, Hyogo, Japan, 26 days after vaccination. The thyroid gland was diffusely enlarged without tenderness on palpation. The thyroid function test revealed elevated free T4 of 4.08 ng/dL (normal, 0.9–1.7) and free T3 of 7.30 pg/mL (normal, 2.3–4.0), with a suppressed thyroid-stimulating hormone (TSH) level of less than 0.005 µIU/mL (normal, 0.61–4.23). Antithyroglobulin antibody (TgAb) and antithyroid peroxidase antibody (TPOAb) were positive, while antibodies to the TSH receptor (TRAb) were negative. Thyroid ultrasonography (USG) showed a heterogeneous, hypoechogenic, enlarged thyroid gland with an estimated thyroid volume of 27.3 mL and normal Doppler flow. Thyroid scintigraphy (^131^I) showed a marked decrease in 3-h uptake rate to 1.3% (normal uptake: 5–15%). Based on the clinical symptoms and laboratory examinations, the patient was diagnosed with PT. She was followed up closely without therapy, and she did not receive the second dose of the vaccine. When she visited our hospital 7 weeks after being vaccinated, she was relieved of the palpitations and her laboratory data had improved, as follows: free T4 was 1.86 ng/dL, free T3 was 3.75 pg/mL, and TSH was 0.010 µIU/mL. Fourteen weeks post-immunization, she had mild hypothyroidism, and after 5 months, her thyroid function returned to normal without treatment. The patient’s thyroid function tests during follow-up are shown in Table 1.

**Table 1:** Patient’s thyroid function tests during follow-up

	Case1	Case2
At diagnosis	(26th day)	(10th day)
TSH (0.61–4.23 µIU/mL)	< 0.005	0.01
FT4 (0.9–1.7 ng/dL)	4.08	2.35
FT3 (2.3–4.0 pg/mL)	7.3	5.42
TPOAb (0–28 IU/mL)	350	< 16
TgAb (0–40 IU/mL)	299	430
TRAb (0–2 IU/L)	1.16	0.98
On follow-up	(7th week)	(5th week)
TSH ( 0.61–4.23 µIU/mL)	0.01	0.005
FT4 (0.9–1.7 ng/dL)	1.86	1.74
FT3 (2.3–4.0 pg/mL)	3.75	4.13
On follow-up	(14th week)	(2nd month)
TSH (0.61–4.23 µIU/mL)	9.42	2.62
FT4 (0.9–1.7 ng/dL)	0.81	1.03
FT3 (2.3–4.0 pg/mL)	2.54	2.66
on follow-up	(5th month)	
TSH (0.61–4.23 µIU/mL)	1.94	
FT4 (0.9–1.7 ng/dL)	1.45	
FT3 (2.3–4.0 pg/mL)	3.07	
	(Days after vaccination)	

Abbreviations: *TSH* thyroid-stimulating hormone, *FT4* free thyroxine, *FT3* free triiodothyronine, *TPOAb* antithyroid peroxidase antibody, *TgAb* antithyroglobulin antibody, *TRAb* antibodies to TSH receptor

## Case 2

**Fig. 1:**
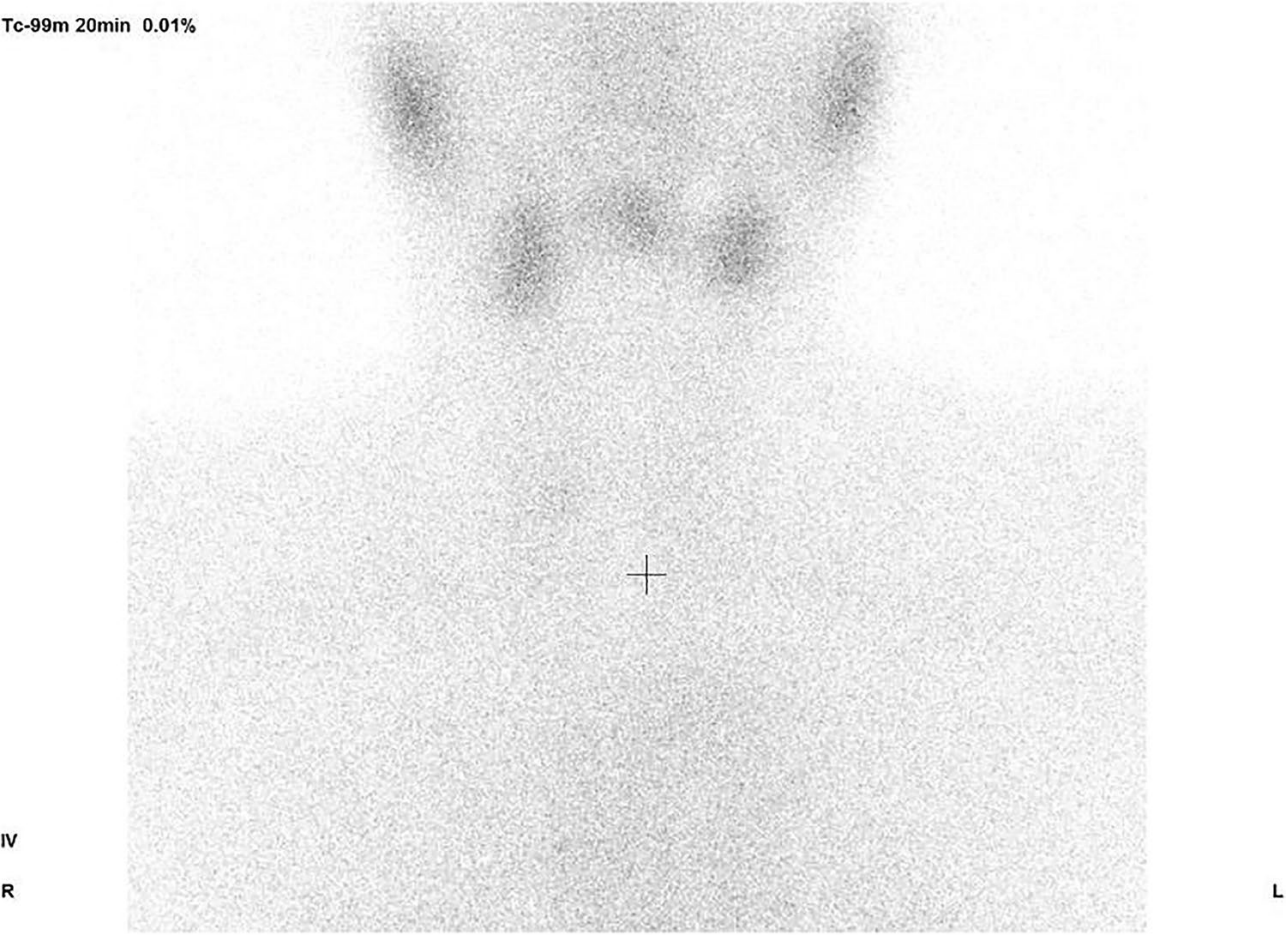
20-min technetium-99 m thyroid scintigraphy of case 2. Thyroid gland uptake was significantly reduced to 0.01%

The patient was a 59-year-old woman who received her second dose of the Pfizer-BioNTech mRNA vaccine for COVID-19 in July 2021. Ten days later, abnormal thyroid function was incidentally detected on a blood test. She was asymptomatic, and her physical examination results were unremarkable. The blood levels of thyroid function were as follows: free T4, 2.35 ng/dL; free T3, 5.42 pg/mL; and TSH, 0.01 µIU/mL. TgAb was positive, and TPOAb and TRAb were negative. The thyroid USG examination revealed that the estimated thyroid volume was 17.9 mL, while the thyroid gland was bilateral, heterogeneous, and hypoechoic with normal blood flow. Her 20-min technetium-99 m thyroid scintigraphy uptake was significantly reduced to 0.01% (normal uptake: 0.5–3.0%) (Fig. 1), and she was subsequently diagnosed with PT. Five weeks after immunization, she was asymptomatic and her laboratory data had slightly improved. Two months post-vaccination, her thyroid function had normalized without medication. The laboratory findings are summarized in Table 1.

To the best of our knowledge, there have been few reports of PT after mRNA vaccination for COVID-19 [2, 4]. PT is regarded as a variant form of Hashimoto’s thyroiditis (HT) because the histopathology of PT is similar to that of HT. Moreover, PT frequently displays high levels of antithyroid antibodies, while many PT patients have a family history of thyroid autoimmune disease.

In such cases, the thyroid gland is non-tender and painless. PT is characterized by transient thyrotoxicosis caused by destruction of the thyroid gland, resulting in excess levels of thyroid hormone [5]. This thyrotoxicosis improves spontaneously and is often followed by a hypothyroid phase and complete recovery.

PT is considered to be an immune-mediated disorder and, as has been reported, may be caused by immune checkpoint inhibitor drugs, with various cytokines, such as interferon-alpha and interleukin-2, being postulated to induce PT.

The condition of postpartum and cessation of glucocorticoids after adrenalectomy in patients with Cushing’s syndrome, as well as other factors, are also reported to cause PT. Overall, it is assumed that these conditions cause thyroid inflammation and damage to the thyroid follicles, resulting in destructive thyroiditis [5].

Various vaccines to prevent severe acute respiratory syndrome coronavirus 2 (SARS-CoV-2) infection have been developed and are available for use. Some contain such adjuvants as aluminum hydroxide, toll-like receptor agonists, and potent Matrix-M1, among others, to enhance the immune response. However, these adjuvants can cause adverse immune reactions known as autoimmune/inflammatory syndrome induced by adjuvants (ASIA syndrome). The cases of subacute thyroiditis following inactivated COVID-19 vaccination have also been reported as an ASIA syndrome [3].

The Pfizer-BioNTech COVID-19 vaccine is an mRNA vaccine that does not contain any of these adjuvants. Nevertheless, cases of autoimmune thyroiditis meeting the criteria for ASIA syndrome have been reported after the use of this vaccine [4], suggesting that a component, or components, of the mRNA vaccine may serve as an adjuvant.

The mRNA vaccine has the potential to prevent SARS-CoV-2 infection by producing a neutralizing antibody against the SARS-CoV-2 spike protein. However, the SARS-CoV-2 spike protein and TPO are structurally similar, and antibodies developed against SARS-CoV-2 may promote autoimmune thyroiditis [6]. Indeed, the case of a 13-year-old girl with a significant increase in TPO antibodies after Pfizer-BioNTech COVID-19 vaccination has been reported [7]. Thus, although there is a lack of definitive evidence, we suggest that PT could be caused by the autoimmunity triggered by the COVID-19 vaccine.

In conclusion, clinicians should be aware that PT may develop after the mRNA COVID-19 vaccination.
